# Functional imaging of brain organoids using high-density microelectrode arrays

**DOI:** 10.1557/s43577-022-00282-w

**Published:** 2022-06-30

**Authors:** Manuel Schröter, Congwei Wang, Marco Terrigno, Philipp Hornauer, Ziqiang Huang, Ravi Jagasia, Andreas Hierlemann

**Affiliations:** Bio Engineering Laboratory, Department of Biosystems Science and Engineering, ETH Zurich, Basel, Switzerland; F. Hoffmann-La Roche Ltd., Roche Innovation Center Basel, Basel, Switzerland; F. Hoffmann-La Roche Ltd., Roche Innovation Center Basel, Basel, Switzerland; Bio Engineering Laboratory, Department of Biosystems Science and Engineering, ETH Zurich, Basel, Switzerland; EMBL Imaging Centre, European Molecular Biology Laboratory (EMBL), Heidelberg, Germany; F. Hoffmann-La Roche Ltd., Roche Innovation Center Basel, Basel, Switzerland; Bio Engineering Laboratory, Department of Biosystems Science and Engineering, ETH Zurich, Basel, Switzerland

## Abstract

Studies have provided evidence that human cerebral organoids (hCOs) recapitulate fundamental milestones of early brain development, but many important questions regarding their functionality and electrophysiological properties persist. High-density microelectrode arrays (HD-MEAs) represent an attractive analysis platform to perform functional studies of neuronal networks at the cellular and network scale. Here, we use HD-MEAs to derive large-scale electrophysiological recordings from sliced hCOs. We record the activity of hCO slices over several weeks and probe observed neuronal dynamics pharmacologically. Moreover, we present results on how the obtained recordings can be spike-sorted and subsequently studied across scales. For example, we show how to track single neurons across several days on the HD-MEA and how to infer axonal action potential velocities. We also infer putative functional connectivity from hCO recordings. The introduced methodology will contribute to a better understanding of developing neuronal networks in brain organoids and provide new means for their functional characterization.

## Introduction

Organoids represent attractive 3D model systems to study the mechanisms of multi-cellular self-organization, such as aspects of mammalian brain development, *in vitro*.^1^ Although it has been known for some time that pluripotent stem cells (PSCs) can develop neuronal properties *in vitro*,^2^ the full potential of today’s organoid technology became apparent after studies demonstrated that PSCs, cultured under appropriate conditions, can form polarized neuroepithelial structures,^3^ which enabled the generation of layer-specific neurons^4^ and even sub-domain-specific brain organoids.^5–10^ However, despite recent evidence that some cellular and morphological developmental milestones of early corticogenesis can be recapitulated in brain organoids, their functional maturation and synaptic connectivity are not yet well characterized.11 Hence, methodology to characterize these properties will be critical in order to probe their relevance in studying human brain development and diseases associated with synaptic dysfunction and altered neuronal activity.

Spontaneous electrical activity during prenatal and early postnatal development is considered to play an important role in the maturation of the cerebral cortex.^12^ Studies using primary cells or organotypic brain slices have demonstrated that distinct patterns of spontaneous neuronal activity represent functional hallmarks of neuronal network maturation.^13^ In rodents, sparse intrinsic calcium activity among small numbers of cells has been observed at embryonic time points,^14^ followed by more coherent activity among gapjunction-coupled ensembles around birth^15^ and early synapse-driven activity patterns in the first postnatal weeks.^16^ Early spontaneous activity is thought to be involved in a broad range of developmental processes, including neurogenesis and migration, as well as the formation and refinement of synaptic connectivity.^17^ Although some studies have begun to demonstrate that brain organoids show orchestrated calcium transients,^6,18–21^ a more detailed characterization of the endogenous electrical activity of human brain organoids during maturation is required.

Microelectrode arrays (MEAs) have commonly been used to study physiological aspects of neuronal populations *in vivo* and *in vitro*, ^22^ but scientists have only recently started to apply MEAs to human brain organoids.^19,23^ However, using passive MEAs with relatively few electrodes (<100 electrodes per mm^2^) and large inter-electrode spacing, these studies could only assess population activity. In contrast, complementary metal-oxide-semiconductor (CMOS)-based high-density MEAs (HD-MEAs) feature high spatiotemporal-resolution readout capabilities, which allow for an efficient de-mixing of neuronal activity that enables single-unit analysis. HD-MEAs, therefore, provide high-resolution access to a variety of neurophysiological features across scales that originate from subcellular compartments, individual neurons, or ensembles of several hundreds of cells over extended time periods.^24–26^

In this proof-of-concept study, we report methods to culture human cerebral organoid (hCO) slices for several weeks on HD-MEAs and to characterize their electrophysiological properties at the single-cell and network level. Using large-scale electrophysiological recordings, we describe how individual neuronal units of sliced hCOs can be tracked over several days and how HD-MEAs can be used to study intricate features, such as axonal action potential (AP) propagation velocity. Moreover, we demonstrate that HD-MEA recordings allow us to probe the endogenous electrical activity of hCOs by pharmacological means and to study their emerging population dynamics and functional connectivity.

## Results and discussion

### Interfacing hCO slices with HD-MEAs

**Figure 1:**
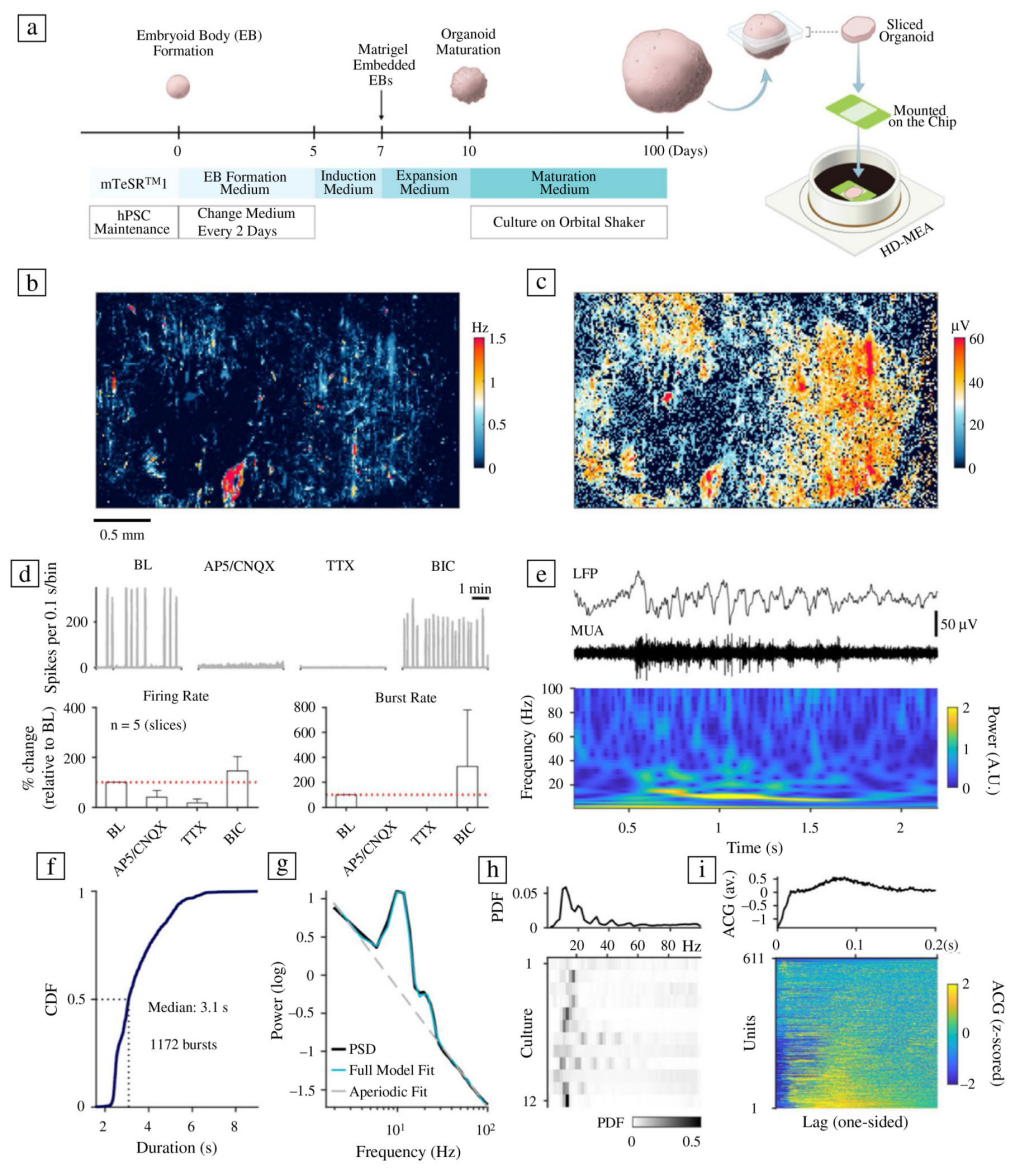
Functional network recordings from human cerebral organoids using high-density microelectrode arrays. (a) Schematic representation of the human cerebral organoid (hCO)-generation protocol. Three-months-old hCOs were sliced and then plated on high-density microelectrode arrays (HD-MEAs). (b) Whole-array HD-MEA activity scan of a sliced hCO two weeks after plating. The spontaneous activity was recorded sequentially in blocks across the full array (2 min per recording configuration, each comprising 1024 recording electrodes). Colours indicate the average multi-unit activity (MUA, Hz); light red/orange colours correspond to higher activity, light and dark blue colours indicate electrodes with lower activity. Scale bar: 0.5 mm. (c) Whole-array spike-amplitude map of the same organoid slice. Orange/red colours indicate electrodes with high amplitude spike activity. (d) Pharmacological perturbation of hCO spontaneous activity with NMDA (AP5), AMPA (CNQX), and sodium-channel blockers (TTX). The upper left panel shows the baseline (BL) activity summed over all active channels in 0.1 s bins (grey trace); the lower panel depicts the group-level data. Sequential application of CNQX and AP5 drastically reduced the overall activity (middle left panel), and activity was abolished by addition of TTX. Application of the GABA_A_ receptor blocker bicuculline (BIC) alone increased the overall activity (panel on the right) and the number of network bursts. Pharmacological recordings were obtained at least 5 min after application of the drug. (e) hCOs demonstrated a range of network burst patterns, including activity that resembled spindle bursts. The two upper panels depict the wideband extracellular local field potential (LFP) signal (1−100 Hz) and the aggregated MUA (0.5−3 kHz) during a burst. The panel below shows a time−frequency scalogram, inferred by a continuous wavelet transform. (f) Cumulative distribution function (CDF) for the burst duration across all bursting hCOs (*n* = 12 slices; the median duration was 3.1 s). (g) The power spectral density (PSD) for hCO oscillatory activity measured during network bursts using methodology that parametrizes the power spectrum into an aperiodic component and oscillatory peaks. (h) Distribution of oscillatory peak frequencies across all bursts and all hCOs (upper panel). The lower panel depicts the burst peak frequency distribution for each hCO separately. (i) The z-scored autocorrelograms for 611 spike-sorted units (pooled over *n* = 14 hCO slices; the colour bar is clipped to −/+ 2).

**Figure 2:**
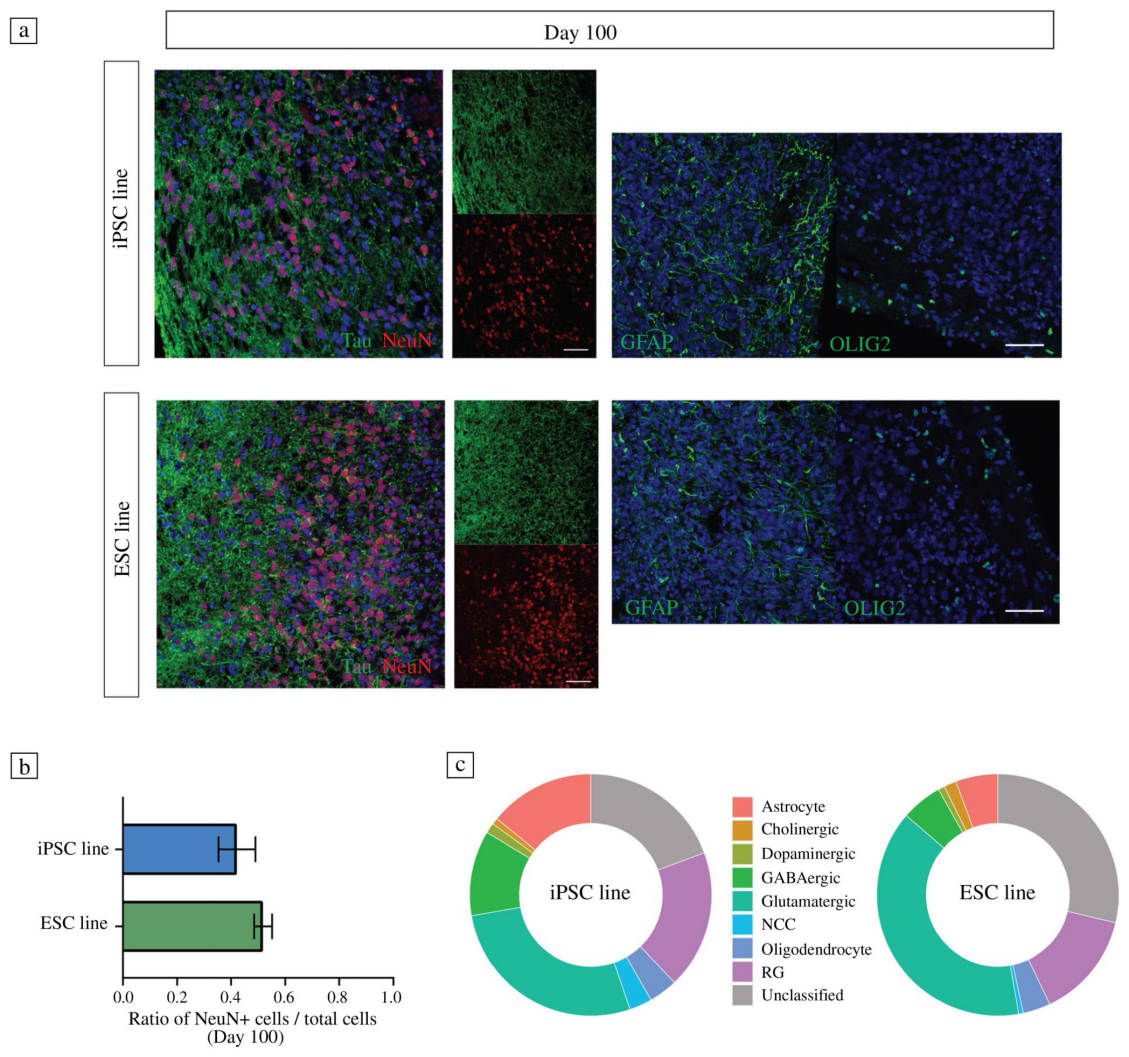
Human cerebral organoids comprise diverse cell types relevant to the study of neuronal circuit development and physiology. (a) Immunohistochemistry (IHC) revealed the presence of mature neurons (Tau/NeuN), astrocytes (GFAP), and oligodendrocytes (OLIG2) after 100 days of differentiation; nuclei were stained with DAPI (in blue). Scale bars are 50 μm. (b) Quantification of NeuN-positive cells. The ratio of the number of NeuN-positive cells to the total number of cells was quantified for both hCO lines (3 hCOs per line, for each hCO > 5000 cells). The ratio of NeuN-positive cells to total cells did not differ significantly between the two lines. (c) Single-cell RNA sequencing (scRNA-seq) was performed to quantify the cellular composition of hCOs and their comparability. The results are summarized in two pie charts with the identified cell type fractions displayed for each line. For both lines the results confirmed the presence of dopaminergic, GABAergic, glutamatergic and cholinergic neurons, neural crest cells (NCCs), radial glia (RG), astrocytes and oligodendrocytes.

Human cerebral organoids (hCOs) were generated from the Gibco human episomal iPSC line and the Takara Bio human embryonic stem cell line, respectively, using the STEMdiff cerebral organoid kit (a commercial kit, based on the protocol by Lancaster et al.^18^; see Materials and methods; Figure 1a). hCOs were collected and characterized by immunohistochemistry (IHC) at two time points, namely, day 50 and day 100. IHC stainings of hCOs at day 50 served as a quality control check (Figure S1), and showed the presence of small neural rosettes containing well-structured progenitor zones (Pax6/Sox2) and neuronal populations (MAP2/Tuj1). Stainings for Tbr1 and Ctip2 revealed the presence of deep-layer cortical neurons. The majority of neurons in the hCOs were stained positive for FoxG1, indicating their forebrain identities (Figure S1). One-hundred-day-old organoids expressed high levels of post-mitotic neuronal markers including Tau and NeuN (Figure 2a); astrocyte (GFAP) and oligodendrocyte (OLIG2) expression confirmed the presence of glia and the maturation of hCOs. After 100 days of culture, we quantified the total neuron population of hCOs using the pan-neuronal marker NeuN (Figure 2b). We found that a significant portion of cells (40−50%) stained strongly for NeuN; the fraction of neurons was comparable for iPSC- and ESC-derived organoids. To further characterize the cell types within hCOs, we performed single-cell RNA sequencing (scRNA-seq) by pooling five to six 100-day-old hCOs per line. Our analysis revealed multiple cell clusters/types by comparing unique transcripts expressed specifically in each cluster, including, but not limited to different neuronal subtypes (iPSC line/ESC line): glutamatergic (67%/83%, *n* = 1650/1683), GABAergic (28%/12%, *n* = 683/234), dopaminergic (3%/2%, *n* = 82/37), and cholinergic neurons (2%/4%, *n* = 51/72). Moreover, there were clusters for neural crest cells (NCC), radial glia (RG), astrocytes, and oligodendrocytes (Figure 2c, Figure S2). In summary, our results are consistent with previously published data,^18^ and underlined that 100-day-old hCOs have reached a certain maturational stage.

To electrophysiologically characterize organoids, we sliced iPSC and ESC-derived hCOs (approximately two slices per organoid, each approximately 500 μm thick) and plated the slices on planar high-density microelectrode arrays (HD-MEAs),^26,27^ adapting procedures previously introduced for organotypic slice recordings.^28^ The dataset included 14 slices, derived from eight different organoids (four organoids of each cell line). hCO slices were plated on the HD-MEA electrode area, freshly coated with Matrigel and fixed with a tissue harp. The developed protocol enabled us to record from sliced hCOs for at least four weeks; the main analyses are based on recordings from hCO slices approximately 2−3 weeks after plating.

### Probing electrical activity of hCOs with HD-MEAs

HD-MEA electrophysiological recordings of sliced hCOs started 3−4 days after plating. To screen for spontaneous neuronal activity, we acquired a series of recording blocks covering the entire HD-MEA sensing area (3.85 x 2.10 mm^2^; 26,400 electrodes of which 1024 electrodes could be connected to read-out channels and simultaneously recorded from). Figure 1b and c depicts the result of an activity scan, with 2-min recordings for a total of 28 configurations, providing a detailed overview of the position and multi-unit spike activity of the hCO. Most slices covered almost the entire sensing area of the chip (> 5 mm^2^). Although not all hCOs showed activity on the first recording days, spiking reliably emerged over the first week in all slices studied.

The recorded activity was a mixture of more regular spiking and occasional large network bursts (Figure 1d, upper panel on the left). Across all included samples in the study (*n* = 14 slices, including both cell lines), 12 hCOs (85%) developed strong network bursts. The burst rate for these 12 organoids was 0.01 ± 0.01 Hz; the average burst duration was 4.05 ± 1.18 s (mean ± S.D.; average for 25.5 h of HD-MEA network recordings, acquired 2−3 weeks after plating). hCOs generated from the ESC line showed a higher burst rate (p < 0.01; Wilcoxon rank-sum test), but a lower burst duration than the iPSC line (p < 0.05; see Figure S3). The average firing rate of all units was 0.38 ± 0.80 Hz (average over 611 spike-sorted units across *n* = 14 hCO slices).

The application of the N-methyl-D-aspartate (NMDA) and α-amino-3-hydroxy-5-methyl-4-isoxazole-propionic-acid (AMPA) receptor antagonists 6-cyano-7-nitroquinoxaline-2,3-dione (CNQX) and D-(-)-2-amino-5-phosphonopentanoic acid (AP5) strongly reduced the measured multi-unit activity (40.66 ± 26.53% compared to baseline (BL), *n* = 5 slices from two batches), which was even more reduced after adding the sodium-channel blocker tetrodotoxin (TTX; 16.62 ± 16.17% compared to BL). The effect of NMDA and AMPA receptor antagonists on hCO activity occurred very consistently across all tested hCOs and blocked network bursts. The GABA_A_ receptor antagonist bicuculline (BIC) caused a moderate increase in hCO multi-unit activity (144.54 ± 58.00% compared to BL, *n* = 5 slices); however, the effect varied quite strongly between samples. The observed variability could be related to the mixed composition of excitatory and inhibitory neurons in hCOs,^29^ as well as their maturity.^30^ Overall, our results are consistent with the results of a previous study^23^ and indicate that neuronal network activity is likely driven by functional excitatory and inhibitory neurons.

Power spectral density (PSD) estimates for the local field potential (LFP) during network bursts were inferred by applying a spectral parametrization method31 (Figure 1g->h). Parametrized PSDs were probed for power peaks in the 2−100 Hz frequency range for the local LFP, i.e., the average signal over small groups of spatially adjacent electrodes (see Materials and methods). Significant peak frequencies were pooled across all LFPs and burst events to assess their likelihood per hCO. Figure 1h shows the detected peak frequency distributions for all bursting hCOs (lower panel, *n* = 12 slices). The group average for the most likely peak frequencies during network bursts was 14.33 ± 4.16 Hz. The observed oscillatory dynamics resembled spindle bursts, previously described in neonatal rodent cortical slices *in vitro*.^32^

### Electrical footprints and action potential waveform clustering of hCO neurons

**Figure 3:**
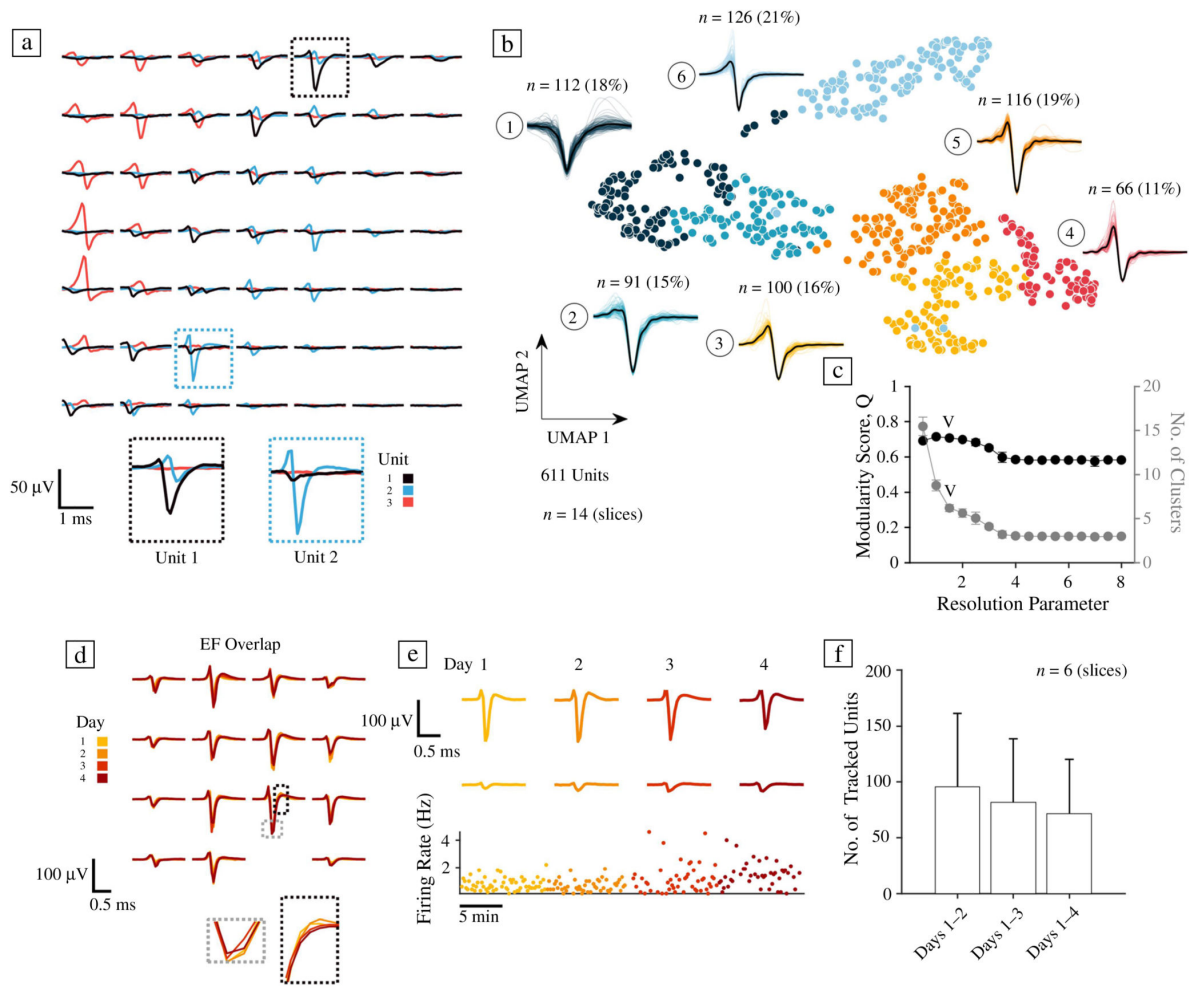
High-density microelectrode recordings allow action potential waveform characterization and long-term tracking of single neurons in human cerebral organoids. (a) Spike sorting of high-density microelectrode array (HD-MEA) recordings of human cerebral organoids (hCOs) enabled analyses of extracellular electrophysiological features at the single-unit level. Three example units, extracellularly recorded from an hCO on a 7 x 7 electrode HD-MEA grid (the centre-to-centre electrode distance is 17.5 μm), are depicted in (a). Each unit has an “electrical footprint” (EF) on the chip, which was inferred by spike-triggered averaging using spike-sorted single-unit activity. The two insets depict the peak channel of unit 1 (in dark blue, on the left) and unit 2 (in light blue, on the right). (b) Uniform manifold approximation and projection (UMAP) and Louvain clustering analysis of high-pass-filtered (> 150 Hz) EF peak channel signals. This panel depicts the similarity of inferred normalized waveforms across all units in the UMAP space; each colour depicts the cluster membership of each unit. Next to each cluster, all waveforms belonging to that cluster and an average waveform (in black) are shown. Waveforms are clipped to 4 ms. The clustering result shows a spectrum, ranging from somatic, monophasic, negative-first-peak channels (on the left) to more axonal waveforms (on the right). (c) Comparison of Louvain modularity score Q and the number of inferred clusters across a range of resolution parameters (average over 20 iterations). The resolution parameter for the clustering analysis in (a) was 1.5. (d) hCO EFs could be used to track individual neurons over several days on the HD-MEA. The depicted template waveform changed very little across the four recording days (see insets). Line colours indicate the different recordings on days 1−4 (from yellow to red). (e) Two example electrode signals of the template in (d) over the four-day tracking period (10 min recording for each day). (f) The number of units that could be tracked over the four-day period decreased with time (*n* = 6 slices; data depicted as the mean ± S.D.).

To investigate the electrophysiological properties of hCO slices at the level of individual neurons, we spike-sorted HD-MEA data and inferred so-called “electrical footprints” (EFs). An EF is the AP-triggered extracellular signature of a single neuron across the electrodes of the HD-MEA^33–35^ (see Figure 3a). Spike sorting was generally challenging for hCO recordings due to their burst-dominated activity and comparably small extracellular spike amplitudes. Following a post-processing step to exclude units with refractory-period violations, potential duplicate spikes, and split units, we studied 611 units (*n* = 14 slices; spike sorting was performed on >1-h-long network recordings, 2−3 weeks after plating).

To quantitatively characterize the extracellularly recorded electrical compartments of hCO EFs, we applied a published AP waveform clustering pipeline.^36^ Here, nonlinear dimensionality reduction via universal manifold approximation and projection (UMAP) was applied to extracellular AP waveforms before using the Louvain community detection algorithm^37^ to find clusters in the high-dimensional graph produced by UMAP. Clustering of hCO EFs was performed on normalized peak channel waveforms, that is, the signal of the electrode featuring the highest amplitude within each EF. Figure 3b depicts the result of a waveform clustering analysis at a resolution parameter that optimized the modularity score (Q) while avoiding the creation of too many small clusters (resolution parameter: 1.5; see Figure S4 for other resolution parameters). As previous HD-MEA studies have documented in 2D neuronal cultures,^38,39^ we found that hCO recordings contained a mixture of signals that likely originated either from axons (signals with bi- or tri-phasic waveforms) or electrical compartments closer to the soma, such as the axonal initial segment (AIS). The clustering analysis revealed that at least three groups, clusters 4 (11%), 5 (19%), and 6 (21%), comprised signals with axonal characteristics (e.g., bi- or triphasic waveforms), that is, more than 50% of all units. Cluster 1 (18% of all units) had wide, monophasic or biphasic negative first waveforms that likely originated from electrodes close to somas. Similarly, cluster 2 (15%) comprised EFs that demonstrated waveforms with a small first peak but otherwise pronounced negative amplitudes, which may originate from signals near the AIS or soma.^38,40^ Future studies may correlate the observed somatic waveform clusters with specific cell types (e.g., to putative excitatory and inhibitory neurons).

### Tracking of single neuronal units over days

We performed a long-term tracking experiment to probe the feasibility of following individual hCO neurons across several recording days (Figure 3d-f; see Reference 41 for a comparable approach in rodent organotypic slices). Using the same electrode configuration with up to 60 high-density electrode blocks, each consisting of 4 x 4 electrodes with a 17.5-μm pitch, we recorded from 6 hCO slices on four consecutive days (recording duration per session: 10 min). The acquired tracking data were then concatenated and spike-sorted. The results show that a substantial number of single-unit EFs could indeed be tracked across days (96 ± 66 EFs from Day 1 to 2, 82 ± 56 across Days 1−3, and 72 ± 48 across Days 1−4), suggesting that the developed protocol is suited for chronic electrical recordings and that individual cells can be tracked. Being able to follow individual hCO neurons over several days could be advantageous for longitudinal electrophysiological studies, such as studies interested in homeostatic plasticity, or long-term alterations in hCO spontaneous activity following drug perturbation.

### Estimating action potential propagation velocity in hCOs

**Figure 4:**
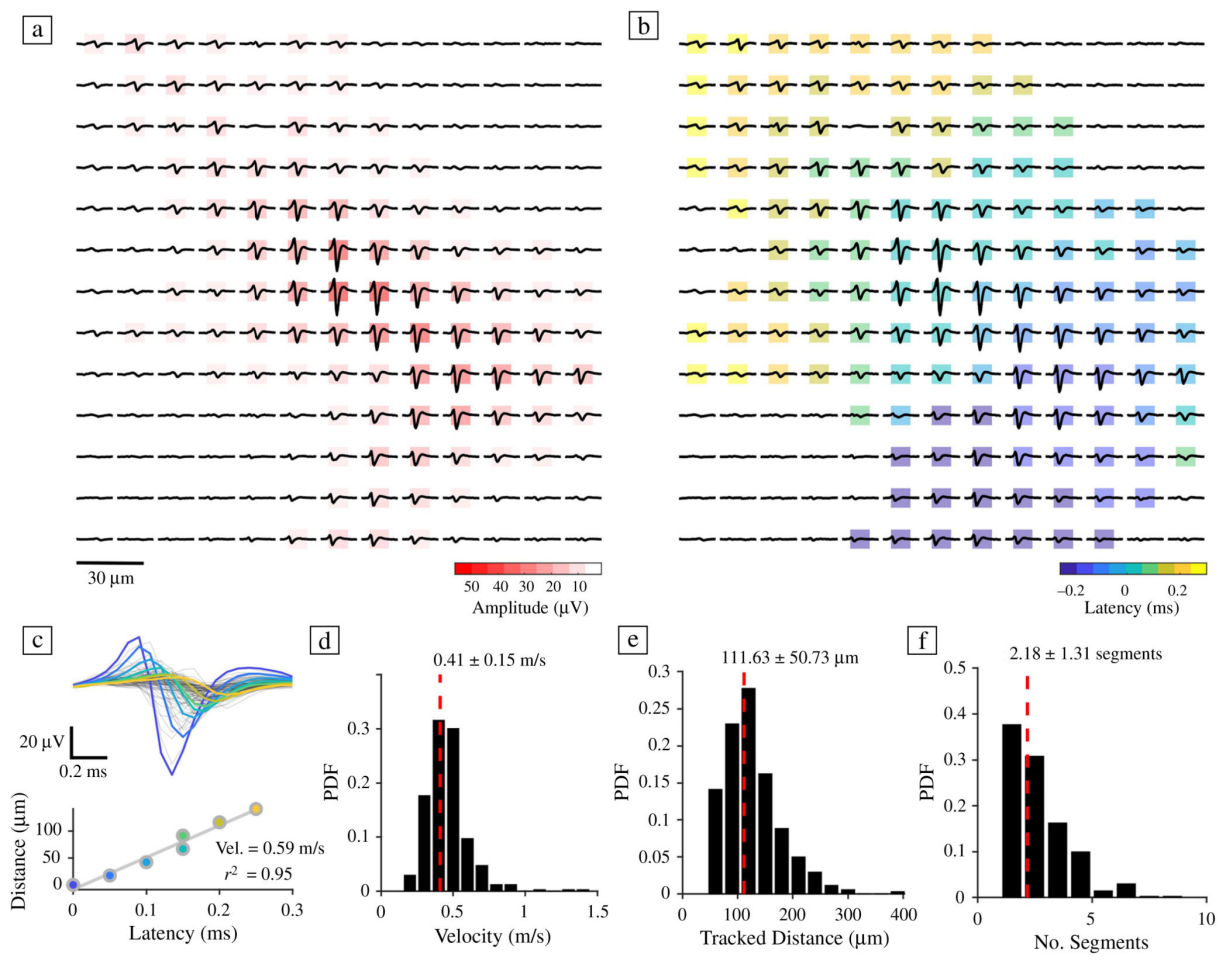
Quantifying action potential propagation velocity in human cerebral organoids. (a) Example electrical footprint (EF) of a human cerebral organoid (hCO) neuron on a high-density microelectrode array (HD-MEA); the electrode centre-to-centre distance is 17.5 μm. Background colours indicate the measured trough amplitude for each electrode (absolute values; in red). (b) The same neuron as in (a), coloured according to the occurrence time of the action potential (AP) peak on each electrode. The latency is depicted relative to the peak time of the electrode with the most dominant trough, the putative location of the axon initial segment (AIS).^38^ Dark blue colours indicate early peak times, yellow indicates electrodes with later AP peak times. (c) Example velocity analysis for the subset of electrodes, whose AP peak times occurred after that of the AIS peak channel. Colours indicate the AP latency of each electrode and correspond to the colours in the regression plot (lower panel). The AP propagation velocity of individual axonal segments was estimated by regressing the AP peak time of each electrode (latency) against the cumulative distances of the propagation path. Each dot corresponds to one electrode. (d) The average propagation velocity was 0.41 ± 0.15 m/s, (e) the averaged tracked AP propagation distance was 111.63 ± 50.73 μm, and the average number of axonal segments per neuron was 2.18 ± 1.31 (f). Group data were pooled across all 14 hCOs. Only units whose *r*^2^ fit values were above 0.8 were included. The dashed red lines in d−f indicate the mean value.

Next, we used sequential HD-MEA block recordings (each block consisted of 20 x 20 electrodes with a 17.5 μm electrode pitch) to infer AP-triggered average EFs and to determine the velocity of axonal AP propagation. Following spike sorting and EF inference, we were able to infer velocity measurements for a total of 339 units (pooled across *n* = 14 slices). Figure 4a−b shows the spatial spread of AP latencies for an example EF and the decay of neuritic AP signal amplitudes with increasing distance from the soma. Figure 4c illustrates the analytical approach to quantify axonal AP propagation velocities from EFs. Axonal AP propagation velocity was estimated by regressing the AP peak times of electrodes, herein referred to as “latencies,” against the cumulative distances of the axonal AP propagation path (Figure 4c). The AP propagation path was inferred by transforming the EF electrode space into a directed-flow graph (each electrode here represents a node) and by searching for the shortest path from the peak channel (AIS) to the most distant electrode of a flow graph cluster (see Materials and methods for details). We included only units that showed a clear temporal ordering of latencies after the EF peak channel (i.e., the channel featuring the most dominant trough), and for which good fitting results could be obtained (*r*^2^ > 0.8). On average, hCOs had an axonal AP propagation velocity of 0.41 ± 0.15 m/s. This value was similar to previously reported values for AP propagation in iPSC-derived human neurons.^42^ The number of inferred EF segments, i.e., putative axonal segments, ranged from 1 to 9, with an average of 2.18 ± 1.31 segments; the average distance that an axonal AP could be traced on the HD-MEA was 111 ± 50 μm (Figure 4e-f). To the best of our knowledge, this is the first time that AP propagation velocities have been inferred from hCOs.

### hCOs demonstrate sparse functional connectivity

**Figure 5:**
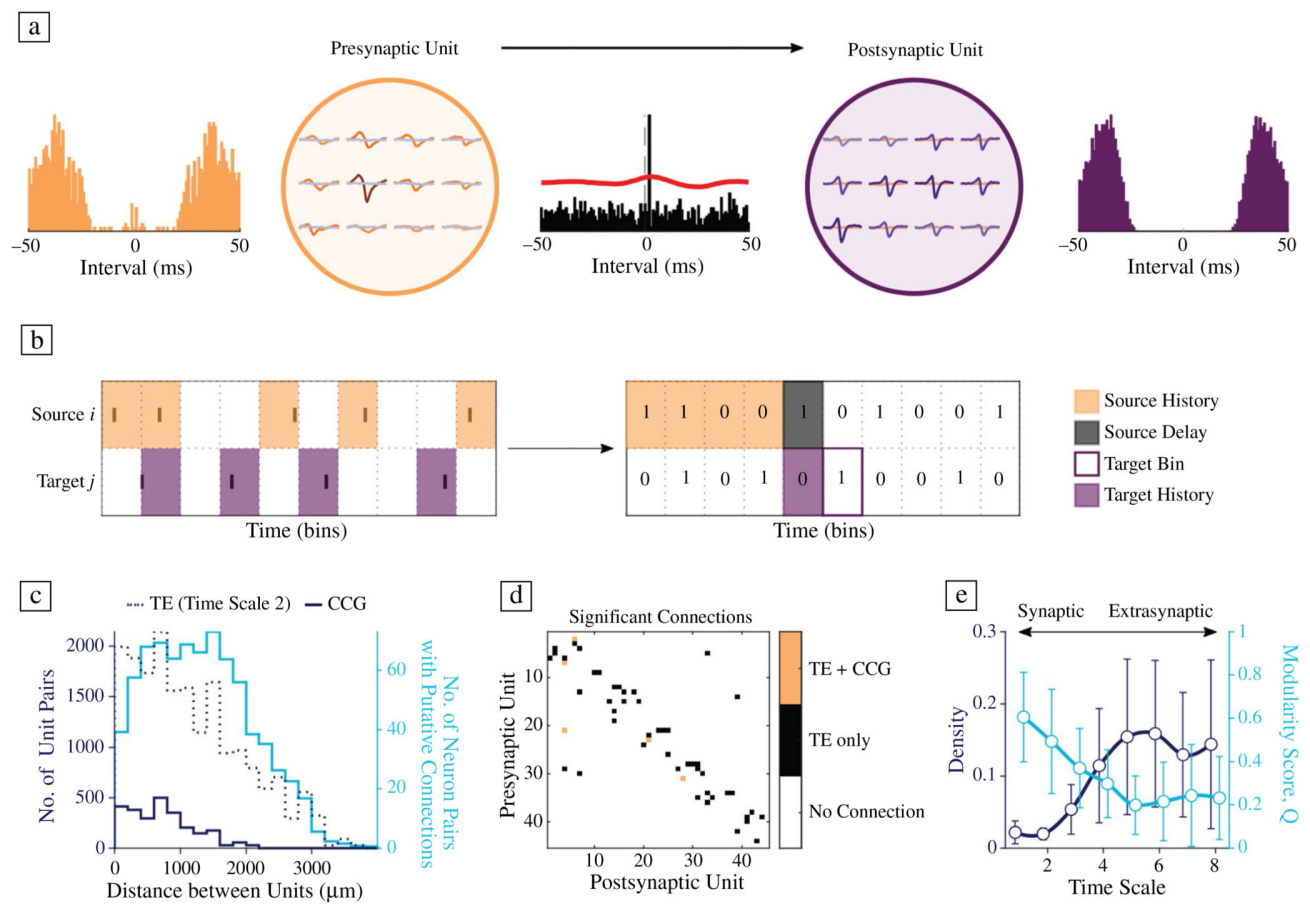
Studying functional connectivity in human cerebral organoids. (a) Example excitatory functional connection as detected by the cross-correlogram (CCG) method. Autocorrelograms of the putative presynaptic unit (in orange, on the left) and postsynaptic unit (in purple, on the right) and their respective electrical footprints (EFs) on the HD-MEA suggest clear separability of the two units. Middle panel: CCG of the two units and the decision boundary (solid red line) that deemed the connection significant. (b) Schematic illustration of the transfer entropy (TE) method to infer functional connectivity. In the first step, spike trains were binned and binarized (presence/absence of a spike in the respective bin). Then, each neuron pair was divided into a source *i* and target *j*. To calculate TE_*i*→*j*_, the source history in a specified bin range after the source delay, together with the target history, was taken into account. (c) Group-level results of the CCG and TE-based functional connectivity inference (data pooled across 14 hCO slices). Short-latency interactions, as detected by the CCG method, were extremely rare (solid dark blue line), yet there was a clear distance decay for the observed significant functional connections. TE-based connectivity was still sparse, but revealed both short- and long-range connections. (d) Overlay of CCG and TE-inferred connectivity matrices for an example hCO. Orange entries indicate connections that were found by both methods (TE and CCG), black entries indicate that these connections were only present in the TE functional connectivity graph. (e) TE inference results across synaptic and extrasynaptic time scales revealed a general increase in connection density at higher time scales (until at least time scale 6, which corresponded to a bin size of 35 ms and a source history of 35−140 ms). The observed increase could correspond to the observed oscillatory dynamics during network bursts (see Figure 1g). The segregation of the TE connectivity graph, here measured by the modularity score Q, decreased for higher time scales.

hCO functional connectivity was inferred using two previously established approaches. For short-latency interactions between neurons (Figure 5a), we searched for significant cross-correlogram (CCG) peaks among the spike-sorted activity of neurons and compared these to their ongoing baseline activity;^43^ (bin size 1 ms; ± 5 ms peak detection window; ± 50 ms lag; > 1-h-long HD-MEA network recordings). Rigorous preprocessing and inspection of whole-array EFs and autocorrelograms of all units allowed us to discern short-range connections between different neurons from signals belonging to the same neuron (e.g., somatic and axonal signals). Connections with very short latencies (< 1 ms) were excluded from our analysis. Across all samples (*n* = 14 slices), only 88 significant connections were detected, highlighting the sparseness of strong connections in developing hCOs. To assess functional connectivity beyond this synaptic interaction window, we quantified the directed information flow between neurons using transfer entropy (TE).^44^ In short, this approach quantifies the information that the spike train of a source (i.e., a putative presynaptic unit) within a specified time window (source history) provides about the spiking activity of a target (i.e., a putative postsynaptic neuron) at a certain point in time (target bin), given its previous activity (target history) (Figure 5b). This approach allowed us to compare the information flow in hCOs at different neurophysiologically relevant time scales by systematically varying the bin size (1−160 ms; see Supplemental Table 1), as previously described.^45^ Comparing the results of the CCG method to the TE results at a comparable time scale (time scale 2, bin size 1.6 ms) revealed differences in the number of significant connections and their decay with increasing distance between neurons (Figure 5c). The CCG method found only connections up to a length of approximately 2 mm, while the TE method detected far more connections overall (*n* = 686), which included longer connection distances of up to 4 mm. Despite differences between the two methods, many of the connections that were picked up by the CCG method were also found by the TE algorithm (Figure 5d). Comparing the TE results across different time scales revealed an increase in the proportion of significant connections (connection density) from 0.02 to 0.16 at time scale 6. After scale 6, the number of significant connections decreased slightly (Figure 5e, blue line). The observed peak at time scale 6 (source window 35−140 ms) may correspond to the peak frequency during observed network burst events (see Figure 1g). The network community structure, quantified by the modularity score (Q) for each TE functional connectivity network, decreased for more extrasynaptic time scales from 0.61 to 0.20 at time scale 5 and plateaued thereafter (Figure 5e, red line).

## Conclusion

Spontaneous electrical activity is a hallmark of early mammalian brain development and is associated with important functional roles in neurogenesis, neuronal migration, and circuit formation.^46^ To what degree human brain organoids recapitulate such activity, and whether the intrinsic neurophysiological properties of developing human brain organoid neurons and circuits can be compared to the maturational changes *in vivo* are still open questions in the field. Here, we presented methods to culture 3- to 4-month-old hCO slices on HD-MEAs and demonstrated that large-scale functional characterization of hCO activity, by means of HD-MEAs, can help to address the physiological relevance of brain organoids at the cellular and network level.

Previous studies have reported the emergence of spontaneous calcium transients and electrical activity in rodent and human brain organoids.^3,19,23,29,30^ In line with these studies, we found that spontaneous network activity of hCOs could be altered by glutamate and GABA_A_ receptor antagonists, indicating the presence of functional excitatory and inhibitory connections. Although the observed activity resembled early network dynamics, as described previously in rodent *ex vivo* slice recordings,^47,48^ further experimental validation across organoid development is needed to better understand its physiological role and the mechanisms underlying its generation. In particular, this holds true for the contribution of GABAergic interneurons to the observed activity.^30^ Different mechanisms at different developmental time points have been described to give rise to early network activity in rodent models, often involving a combination of intrinsic pacemaker properties of specific neurons and transient connectivity;^49,50^ the extent to which these findings can be translated to human brain organoids warrants future studies.

We used spike-sorted HD-MEA recordings to study the emerging neuronal networks in hCOs and found that hCOs developed very sparse functional connectivity. The formation of synaptic connections may coincide with the emergence of more correlated network dynamics^46^ and has been reported for some cortical neurons in post-mortem human foetal brain tissue.^51^ It is important to note, however, that our study used a planar HD-MEA, hence we can only report on inter-neuronal connections of units located near the surface of the hCO slice. Future validation experiments on the biophysical underpinnings of the observed functional connections are needed. For example, studies could match the inferred functional connections to ground-truth data derived from simultaneous HD-MEA patch-clamp recordings^52^ or paired patch-clamp recordings. Moreover, studies should also look into the effect of including units with both somatic and more axonal EF peak channel waveform characteristics on connectivity statistics.

Our results also provide insights into more intricate features of hCO neurons at the cellular level, such as their axonal AP propagation velocity. We found that the obtained velocity measurements are comparable to those in previous reports of human iPSC-derived neurons cultured in 2D on HD-MEAs.^42^ Nevertheless, our approach to infer velocity could be further improved, for example, by single-cell targeted electrical stimulation, which would give us control of the spiking onset,^39,53^ and enable velocity estimates that are independent of the overall activity level. Axon-velocity measurements and features inferred from AP waveform classifications could provide important insights into the development and physiology of hCO neurons, and their axons, and could represent valuable features to assess phenotypic differences between specific organoid lines and cell types.

Recent studies have indicated that sliced organoids can be maintained *in vitro* for extended time periods and that they do provide additional insights into organoid physiology and development.^19,54,55^ Nevertheless, it must be taken into account that developing neurons and early network activity patterns are sensitive to environmental factors and properties of their extracellular milieu.^56^ Although we see some parallels in the spontaneous electrical activity of sliced hCOs in comparison to previous reports on intact organoids,^23^ for example, concerning their response to glutamate and GABAergic pharmacological agents and their burst rate, further investigations regarding the effects of slicing and culture microenvironments are necessary to understand the exact role of the observed network activity. Again, it should be noted, that due to the planar chip architecture of our HD-MEAs, only a fraction of the plated hCO slices could be recorded; measurements from deeper tissue layers within the organoids were not feasible. This limitation also holds for MEA measurements from intact organoids;^23^ hence, the development of more advanced recording technology, enabling 3D high-resolution large-scale electrophysiological measurements in intact organoids, is urgently needed.^57^

Future studies may combine HD-MEA recordings with single-cell transcriptome analyses to provide further mechanistic insights into the link between genetically determined and activity-dependent processes,^58^ as well as the principles of hCO micro-scale neuronal network organization.^59^ HD-MEA-based organoid screenings may further be useful for testing the consequences of alterations in early spontaneous activity as a risk factor for neurodevelopmental disorders.^60^ Epidemiological studies have found that some insults during gestation—induced, for example, by toxins, substance abuse or inflammatory processes—are correlated with neuropsychiatric disorders.^61^ This link could potentially be tested experimentally by HD-MEA organoid electrophysiology and neurotoxicity screens.^62^

In summary, HD-MEAs provide a powerful, multi-purpose platform to address the physiology of developing human neural tissue, enabling parallel recordings from a large number of cells at high spatiotemporal resolution. We believe that the presented platform has enormous potential to model syndromic diseases associated with neuronal development and synaptic biology.

## Materials and methods

### Human iPSC and ESC-derived cerebral organoids

Human induced pluripotent stem cell (hiPSC) and human embryonic stem cell (hESC)-derived cerebral organoids were generated from two commercially available stem cell lines, the hiPSC line (Thermo Fisher Scientific, Waltham, MA, USA, #A18945) and the hESC line (Takara Bio, Osaka, Japan, #Y00060), using the STEMdiff cerebral organoid kit (STEM-CELL Technologies, Vancouver, BC, Canada, #08570) following the manufacturer’s instructions. In brief, on day 0, hiPSCs/ hESCs were resuspended in embryoid body (EB) formation medium, supplemented with 10 μM Y27632 (STEMCELL, #72304) and further seeded into 96-well round-bottom ultra-low-attachment plates (Corning, Corning, NY, USA, #7007) at a density of 9000 cells/well. EB formation medium (100 μl) was added to each well on days 2 and 4. On day 5, EBs were transferred into 24-well ultra-low attachment plates (Corning, #3473) with neural induction medium (kit) for 48 h. EBs were further embedded in Matrigel (Corning, #354277) and grown in neural expansion medium (kit) for 3 days in 6-well ultra-low-attachment plates (Corning, #3471). After day 10, the neural expansion medium was replaced with neural maturation medium (kit). The 6-well plates containing organoids were transferred onto an orbital shaker (INFORS HT, Bottmingen, Switzerland). A full medium change with neural maturation medium was performed every 2−3 days.

### High-density microelectrode arrays

Human cerebral organoids (hCOs) were recorded on single- and multi-well planar high-density microelectrode arrays (HD-MEAs) provided by MaxWell Biosystems (MaxWell Biosystems, Zurich, Switzerland). The single-well complementary metal-oxide-semiconductor (CMOS)-based HD-MEA (“Max-One”) comprises 26,400 platinum microelectrodes (electrode size: 9.3 x 5.3 μm^2^) at a 17.5 μm pitch (centre to centre) within a total sensing area of 3.85 x 2.10 mm^2^.^26,27^ The MaxOne system allows for simultaneous recordings from up to 1024 readout electrodes or channels at a sampling rate of 20 kHz; the electrodes can be flexibly selected and reconfigured according to experimental needs. To decrease the impedance and to improve the signal-to-noise ratio, electrodes were coated with platinum black;^26^ a characterization of the electrode impedance for comparable HD-MEAs^26,27^ has been provided in a previous study.^63^ Additionally, we used the multi-well system by MaxWell Biosystems (“MaxTwo”). This system comprises 6 HD-MEA wells that are equipped with HD-MEAs featuring the same technical details as previously described for the Max-One HD-MEA. Recordings with the MaxTwo system were sampled at 10 kHz. For both setups, the acquisition filter band cut-off was approximately 1 Hz.

### Slicing and plating of hCOs on HD-MEAs

Cross-sectional slices were obtained from 100-day-old hCOs. Single organoids were first transferred from maturation medium to ice-cold BrainPhys (STEMCELL, #05790) using cut 1000-μl pipette tips. Next, cross-sectional 500-μm-thick slices were cut from hCOs using a sterile razor blade and collected in 6-well plates filled with Brain-Phys medium at room temperature. For recovery, slices were housed in a humidified tissue culture incubator for 1 h before plating. Before plating, single- and multi-well HD-MEAs were sterilized in 70% ethanol for 30 min and rinsed 3 times with distilled water. To improve tissue adhesion, arrays were coated with 0.05% (v/v) poly(ethylenimine) (Sigma-Aldrich, St. Louis, MO, USA, #P3143) in borate buffer (pH 8.5, Thermo Scientific, #28341) for 40 min at room temperature, rinsed with distilled water, and left to dry. To attach hCOs to HD-MEAs, we adopted procedures previously developed for organotypic slices:^28^ First, we applied a thin layer of Matrigel (Corning) to the center of the HD-MEA sensing area and then transferred individual organoid slices from the 6-well plates to the coated HD-MEAs. After positioning the tissue, we placed a tissue “harp” on top of the organoid slice and applied several drops of recording medium (STEMCELL, #05793) around the organoid. HD-MEAs were then covered with a lid and placed in a humidified incubator at 37°C and 5% CO_2_/95% air for 30 min. Following the incubation, we carefully added more medium. In the following 1−2 days, the medium was increased to a final volume of 2 ml per chip. Half of the recording medium was changed every 2−3 days.

### Electrophysiological recordings

To probe single-cell and network activity of sliced hCOs and to identify neurons on the HD-MEA, we performed a series of activity scans, i.e., sequential high-density recordings covering all electrodes of the array. To select recording electrodes, the multi-unit activity (MUA) for each electrode configuration was estimated using a sliding-window, threshold-crossing spike detection algorithm (detection threshold: 5 x the root mean square error of the noise of the bandpass-filtered signal). After the activity scan, and depending on the requirements of each analysis (single-cell or network-related features), we selected the readout electrodes based on the online detected activity. HD-MEA recordings of hCO slices started two days after plating. The reported electrophysiological results are based on data obtained from 14 hCO slices, derived from eight organoids (four organoids for each cell line) and maintained for at least 2−3 weeks on the chip. Twelve slices were recorded with two MaxTwo plates; two slices were recorded with a MaxOne chip. Data were pooled across lines and slices for general statistics. The MaxOne and MaxTwo systems are commercially available from Max-Well Biosystems AG, Zurich, Switzerland.

### Pharmacological procedures

Pharmacological methods were applied to test the effect of glutamate and GABA receptor antagonists on hCO spontaneous multi-unit activity (MUA) and network bursts. Specifically, we tested the effect of the AMPA receptor antagonist CNQX (20 μM, Sigma-Aldrich, #C239), the NMDA receptor antagonist D-AP5 (AP5, 20 μM, Abcam, Cambridge, USA, ab120003), the GABA_A_ receptor antagonist bicuculline (BIC, 20 μM, Abcam, ab120107) and the sodium-channel blocker tetrodotoxin (TTX, 1 μM, Tocris, Bristol, United Kingdom, #1069). After a longer baseline (BL) recording (1 h), we removed 100 μl medium from the HD-MEA. This 100-μl volume of medium was mixed with the diluted pharmacological compound and then carefully returned to the HD-MEA for the pharmacological test. After a waiting period of at least 5 min, we started the HD-MEA recording for each drug condition. AMPA/NMDA receptor antagonists and TTX were sequentially added to the medium. Three washes with recording medium (full media exchange) were performed after the AMPA (CNQX)/NMDA (AP5), respectively, the GABA_A_ receptor (BIC) experiments. Spontaneous activity for most hCOs returned to normal levels 2−3 days after washout (data not shown).

### Immunohistochemistry

Human cerebral organoids were fixed in 4% paraformaldehyde (PFA) for 2−4 h at room temperature, washed three times with PBS and then immersed in 30% sucrose solution at 4°C overnight. PFA-fixed organoids were then embedded in optimal cutting temperature compound (Sakura Finetek, Alphen aan den Rijn, Netherlands, #4583), frozen on dry ice and stored at − 80°C. Thin sections (10 μm) were cut on a cryostat and collected on Superfrost Plus microscope slides (Thermo Scientific, #22-037-246). For immunohistochemistry, sections were permeabilized in 0.1% Triton X-100 and blocked with animal-free blocker (Vector Laboratories, Burlingame, CA, USA, #SP-5030-250). Primary antibodies were diluted in blocking buffer and incubated for 1 h at room temperature. Sections were washed in PBS three times and further incubated with secondary antibodies for 1 h at room temperature. After washing three times with PBS, sections were incubated with PureBlu DAPI (Bio-Rad, Hercules, CA, USA, #1351303) for 3 min and mounted with ProLong Gold antifade mounting medium (Thermo Scientific, #P36930). Fluorescence images were acquired with an SP8 confocal microscope (Leica, Wetzlar, Germany). The following primary and secondary antibodies were used in this study: Pax6 (BioLegend, San Diego, CA, USA, #901301, 1:300), Sox2 (Sigma-Aldrich, AB5603, 1:300), FoxG1 (Abcam, ab18259, 1:200), Tuj1 (BioLegend, #801202, 1:800), Ctip2 (Abcam, ab18465, 1:200), Tbr1 (Abcam, ab31940, 1:300), MAP2 (Thermo Scientific, PA1-10,005, 1:800), Tau (Thermo Scientific, MN1000, 1:500), NeuN (Boster Bio, Pleasanton, CA, USA, M11954-3, 1:300), GFAP (Novus Biologicals, Engle-wood, CO, USA, NB300-141, 1:500), goat anti-mouse IgG (H + L) highly cross-adsorbed secondary antibody, Alexa Fluor Plus 488 (Thermo Scientific, A32723, 1:400), goat anti-rabbit IgG (H + L) highly cross-adsorbed secondary Antibody, Alexa Fluor 568 (Thermo Scientific, A11036, 1:400), goat anti-rat IgG (H + L) cross-adsorbed secondary antibody, Alexa Fluor 647 (Thermo Scientific, A21247, 1:400) and goat anti-chicken IgY (H + L) cross-adsorbed secondary antibody, Alexa Fluor Plus 647 (Thermo Scientific, A32933, 1:400).

### Quantification of NeuN-positive cells in hCOs

To measure the NeuN-positive cell fraction in the hCOs, we performed immunohistochemistry as described above. Sections from three different organoids per line were used for quantification. Quantification was performed using a semiautomated cell counting method. For each confocal dataset, we first generated 2D images by averaging Z slices across the stack. StarDist 2D segmentation was applied to the DAPI channel to define the outlines of the nuclei.^64^ The average NeuN signal (fluorescence intensity) was measured. We applied the same threshold (high pass) to all datasets to generate the NeuN-positive cell fraction estimate. All images were processed and analysed by applying the same procedure described. A total of 5,000−10,000 cells per organoid section were sampled.

### Single-cell RNA sequencing

Single-cell RNA sequencing (scRNA-seq) libraries of hCOs were prepared as described^65^ with minor modifications. Briefly, five to six hCOs per line were washed once with 5 ml PBS (Thermo Scientific, #14190144) and cut into small pieces. The pieces were then washed three times with PBS and digested with 5 ml of papain solution, supplemented with DNase, using a Worthington Papain Dissociation System kit (Worthington Biochemical, Lakewood, NJ, USA, #LK003150) at 37 °C for 30 min and pipetting every 10 min to facilitate cell dissociation. Papain was inactivated with 3 mL of ovomucoid protease inhibitor. Dissociated cells were passed through a 30 μm strainer and then centrifuged at 500 x *g* for 5 min. Live cells were counted using trypan blue staining and centrifuged at 500 x *g* for 5 min, and single cells were resuspended in ice-cold PBS at a concentration of 1 million cells/ml. Subsequently, 6000 cells per sample were processed and barcoded with the Chromium Single Cell 3’ Reagent Kit user guide (v3.1 Chemistry) following the manufacturer’s instructions (10 x Genomics, Pleasanton, CA, USA, #1000128).^66^ cDNA libraries were sequenced on an Illumina NovaSeq 6000, and scRNA-seq was demultiplexed and aligned to the GRCh38 human transcriptome with Cell Ranger (V3.0.0) software (10 x Genomics). A total number of 7498 and 5478 cells with a median of 2652 and 2860 genes per cell were analysed for the iPSC and ESC lines, respectively. Post-processing and bioinformatics analysis of the filtered Cell Ranger output matrix was performed using Seurat.^67,68^ Cells with more than 5000 or less than 200 detected genes, as well as those with mitochondrial transcript proportions higher than 15%, were excluded. Cell clustering was performed on the top 20 principal components (PCs) with a resolution of 0.5. Cell types were assigned to the clusters as described previously with modifications.^69–71^ Briefly, we identified the cells as neurons (STMN2, GAP43, and DCX), astroglia (HES1, SOX2, and S100B), NCCs (FOXD3, MPZ, CDH19, and SOX10), MSC (RSPO2, DCN, and BGN), RC (RAX, SIX3, OPN1SW, RCVRN, and TULP1) or CP (TTR and AQP1). We further classified the neurons into glutamatergic (GRIN1, GRIN2B, SLC17A7, SLC17A6, GLUL, and GLS), GABAergic (GAD1, GAD2, SLC32A1, DLX1, DLX2, and DLX5), cholinergic (ACHE, SLC18A3, and CHAT) or dopaminergic neurons (NR4A2, TH). The astroglial cells were further divided into RG cells (PAX6, GLI3, TOP2A, and MKI67), astrocytes (IL33 and ID3) and OL cells (OLIG1 and OLIG2).

### Data analysis

#### Spike sorting

HD-MEA network recordings were spike-sorted using Kilosort 3.^72,73^ The output of Kilosort was loaded into Phy 2.0,^33^ and the quality of the spike-sorting output was further evaluated in various semi-automatic post-processing steps. These steps included the assessment of the waveform similarity between templates, their refractory periods and inspection of the auto- and cross-correlograms of each putative unit. Noisy templates and templates with refractory-period violations (> 5% of all spikes in the 0−1 ms bin) or too little activity were excluded.

#### Inferring electrical footprints

Spike-sorted HD-MEA network recordings were used to infer hCO single-cell electrophysiological features based on so-called “electrical footprints” (EFs). An EF is the extracellular electrical potential landscape associated with a single neuron on the HD-MEA, which is usually obtained by spike-triggered averaging of multiple signals on all electrodes that record signals of a specific unit. The recording electrode and channel with the highest absolute signal amplitude within the EF will be referred to as its “peak channel”. EFs were extracted from the HD-MEA data through spike-triggered averaging and by adopting previously published procedures.^35^

#### Extracellular action potential waveform clustering

Following spike sorting and EF inference, we applied the WaveMAP pipeline,^36^ to probe whether EF peak channel signals could be grouped into extracellular waveform clusters. We therefore pooled all units across all hCO slices (611 units in total, *n* = 14 slices). First, waveforms at 20 kHz were high-pass filtered (150 Hz) and normalized to the trough amplitude. Then, 3 ms snippets of all waveforms (cut to 1 ms before and 2 ms after the AP peak; 61 points in length) were passed to the uniform manifold approximation and projection (UMAP) algorithm^74^ to generate a high-dimensional graph. This high-dimensional graph captures the relationships across all units and is then used for a graph clustering operation with the Louvain community detection algorithm.^37^ The number of clusters resulting from the Louvain community step depends on a resolution parameter. As in the original publication,^36^ we chose a resolution parameter of 1.5 for the main analysis because it maximized the Louvain modularity score Q, while avoiding the creation of too many small clusters. We provide WaveMap clustering results for a range of resolution parameters in the supplemental material (Figure S4).

#### Axonal propagation velocity

The analysis of axonal propagation velocities of hCO neurons was based on data acquired through high-density block-scan recordings across the array (5−6 min per recording block, each block comprising 20 x 20 electrodes; 17.5 μm electrode pitch). After spike sorting and curation of the data, we up-sampled all EFs to 20 kHz, if necessary, and removed electrodes whose signals did not exceed the baseline noise. Then, the relative AP latency of each electrode with respect to the EF peak channel was inferred, and analysis restricted to electrodes with AP latencies, indicating that the recorded signal occurred after that on the peak channel. Finally, we generated a directed flow graph with each electrode representing a node. A directed edge from electrode *i* to *j* was considered if the relative AP latency of *i* was smaller than the latency of *j* and if the distance to the next electrode was ≤60 μm. We then applied a clustering algorithm to the resulting flow graph^75^ and searched for the shortest path from the peak channel to the most distant electrode of each flow graph cluster. Finally, we performed linear regression analyses to estimate axonal velocity as a function of AP latency and cumulative travel distance for each axonal path. Only units with a reliable velocity estimate were included in the analysis (*r^2^* > 0.8). For the clustering analysis, we used algorithms provided by the Brain connectivity toolbox.^76^

#### Inferring functional connectivity

Putative monosynaptic connections in local circuits have been approximated by probing the short-latency dynamics between spike trains.^77^ Here, we applied a previously established approach to infer such short-latency interactions between pairs of neurons using baseline corrected cross-correlograms.^43^ In short, peaks in the cross-correlogram (CCG) between two spike trains that fell within the monosynaptic window (± 5 ms) were compared to the low-frequency baseline that was obtained from convolving the CCG with a Gaussian kernel (convolution window: 10 ms). The probability of the observed peak—given the low-frequency baseline rate—was then estimated using the Poisson distribution and compared to the chosen alpha (α) level. Here, a low alpha threshold was chosen to ensure that only reliable connections were detected (α = 0.001). Prior to the inference, duplicate spikes in close units were removed to prevent distortions in the resulting CCGs, using the post-processing function *remove_ks2_duplicate_spikes* (0.5 ms window, 100 μm radius) provided by Kilosort. CCGs between pairs of neurons were then calculated using binned spike trains (1 ms bin size) within a ± 50 ms window. CCGs that displayed the peak in the first bin (< 1 ms connection latency) were excluded from further analysis.

Functional connectivity was also inferred using transfer entropy (TE), a measure that quantifies the directed information transfer of one time series to another.^44^ Here, we used a toolbox implementation of the bivariate TE to quantify the functional connectivity of each neuron pair.^78^ To measure interactions over several neurophysiologically relevant time scales, we systematically altered the bin size and delays as previously described^45^ (see Supplemental Table 1). Other parameters were left unchanged for all time scales (history_target = 1, history_ source = 4); all spike trains were binarized to ensure comparability across time scales. The resulting TE values were thresholded by comparing the empirical TE value to the TE values of jittered surrogate spike trains. To this end, the source spike train was jittered using a uniform distribution with a width of seven bins, while the target spike train was left unchanged. The empirical TE value was then z-transformed using the mean and S.D. of the surrogate spike train TE values (*n* = 50). If the empirical z-value exceeded the z-value of a Gaussian normal distribution at the chosen α, a putative functional connection between the pair of neurons was assumed. The threshold was set to α = 0.001.

#### Network burst detection and power spectral density analysis

Network burst events were detected on the binned (0.1 s), normalized, and smoothed multi-unit activity (MUA) vector, inferred online from HD-MEA network recordings, using the peak detection function *findpeaks* in MATLAB. The threshold to detect bursts was defined as the mean + 2 x S.D. of the normalized MUA vector. The burst rate was defined as the number of bursts per second (Hz). The burst duration was defined as the time from the burst onset until the burst ceased; onset and offset time were defined by crossings of a lower threshold (mean of the normalized MUA vector). For the pharmacology experiment, we set the detection threshold to a fixed value based on the observed baseline activity.

Power spectral density (PSD) was estimated during burst periods with Welch’s method (*pwelch* function in MATLAB) on the downsampled (1 kHz) local field potential (LFP) signal. PSDs were calculated on the mean LFP signal, averaged over small groups of spatially clustered electrodes (average over 7−8 adjacent electrodes). Dominating oscillatory peak(s) in the power spectrum, referred to as “peak frequencies,” were detected using the *fooof* toolbox.31 The frequency range to detect peaks in the parametrized power spectrum was set to 2−100 Hz; the minimum peak power was set to 0.3.

#### Statistical analysis

Computational and statistical analyses were performed in MATLAB R2019b (The MathWorks, Natick, USA) and Python 3.7. Data are presented as the mean ± S.D. throughout the text if not otherwise stated. The Wilcoxon rank-sum test was used to compare the two cell lines (iPSCs and ESCs).

## Supplementary Material

Supplementary Material

## Data Availability

Code and data are available upon reasonable request to the corresponding author.

## Impact statement

Human cerebral organoids (hCOs) represent an attractive in vitro model system to study key physiological mechanisms underlying early neuronal network formation in tissue with healthy or disease-related genetic backgrounds. Despite remarkable advances in the generation of brain organoids, knowledge on the functionality of their neuronal circuits is still scarce. Here, we used complementary metal-oxide-semiconductor (CMOS)-based high-density microelectrode arrays (HD-MEAs) to perform large-scale recordings from sliced hCOs over several weeks and quantified their activity across scales. Using single-cell and network metrics, we were able to probe aspects of hCO neurophysiology that are more difficult to obtain with other techniques, such as patch clamping (lower yield) and calcium imaging (lower temporal resolution). These metrics included, for example, extracellular action potential (AP) waveform features and axonal AP velocity at the cellular level, as well as functional connectivity at the network level. Analysis was enabled by the large sensing area and the high spatiotemporal resolution provided by HD-MEAs, which allowed recordings from hundreds of neurons and spike sorting of their activity. Our results demonstrate that HD-MEAs provide a multi-purpose platform for the functional characterization of hCOs, which will be key in improving our understanding of this model system and assessing its relevance for translational research.
