# Supplementary material for: Functional imaging of brain organoids using high-density microelectrode arrays
Source: MRS Bull. Author manuscript; Available in PMC 2022 Sep 23. (PMC9474390; doi:10.1557/s43577-022-00282-w)
Supplement: Supplementary Material [file EMS146547-supplement-Supplementary_Material.docx]

## **Supplemental material**

**
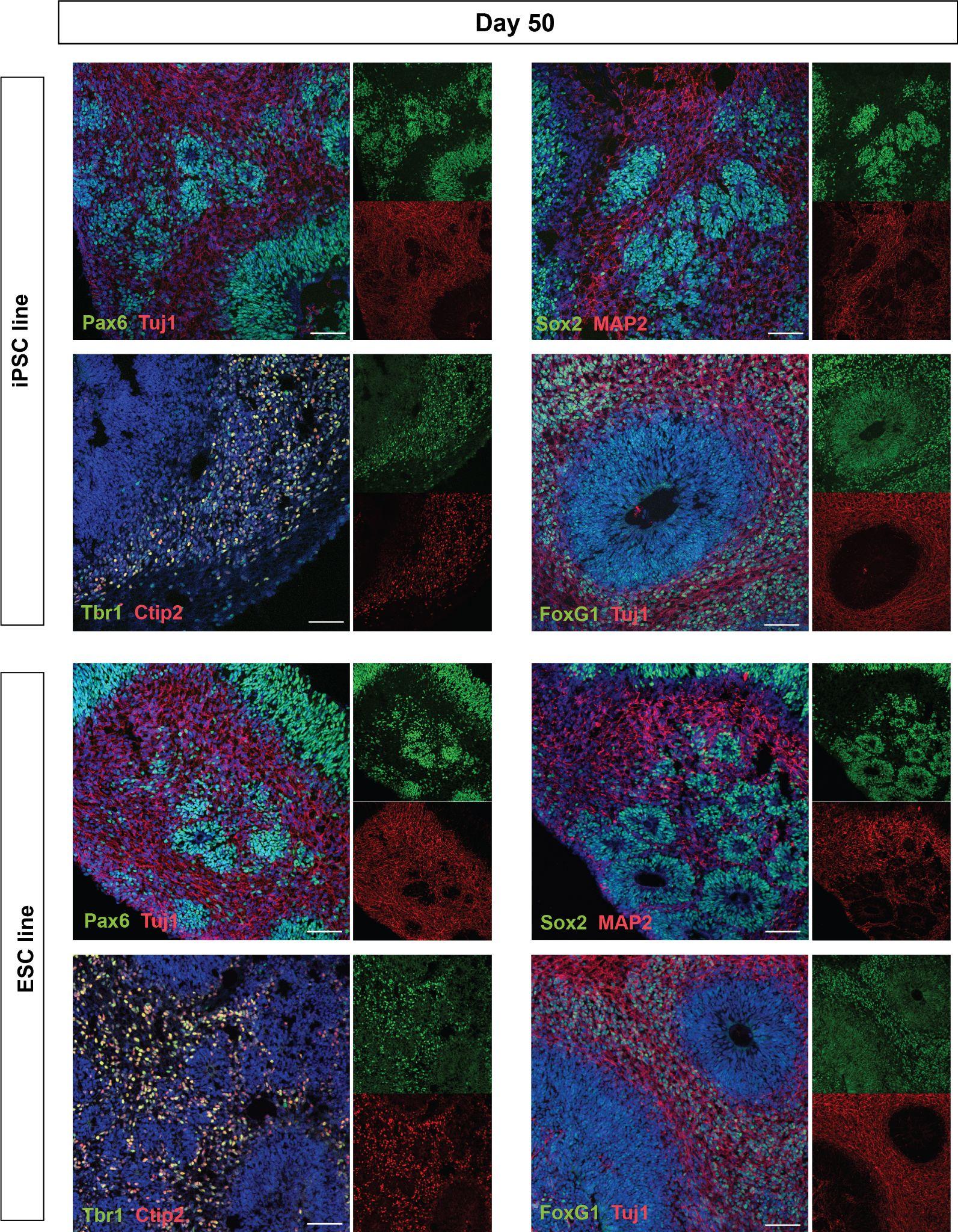
**

**Figure S1. Immunohistochemical characterization of hCOs at day 50.** Immunohistochemistry (IHC) at day 50 of hCO differentiation showed expression of the neural stem cell marker (Sox2), forebrain markers (Pax6/FoxG1), neuronal markers (Tuj1/MAP2) and deep-layer neuron markers (Tbr1/Ctip2). Nuclei were stained with DAPI (blue). Scale bars are 75 μm.

**
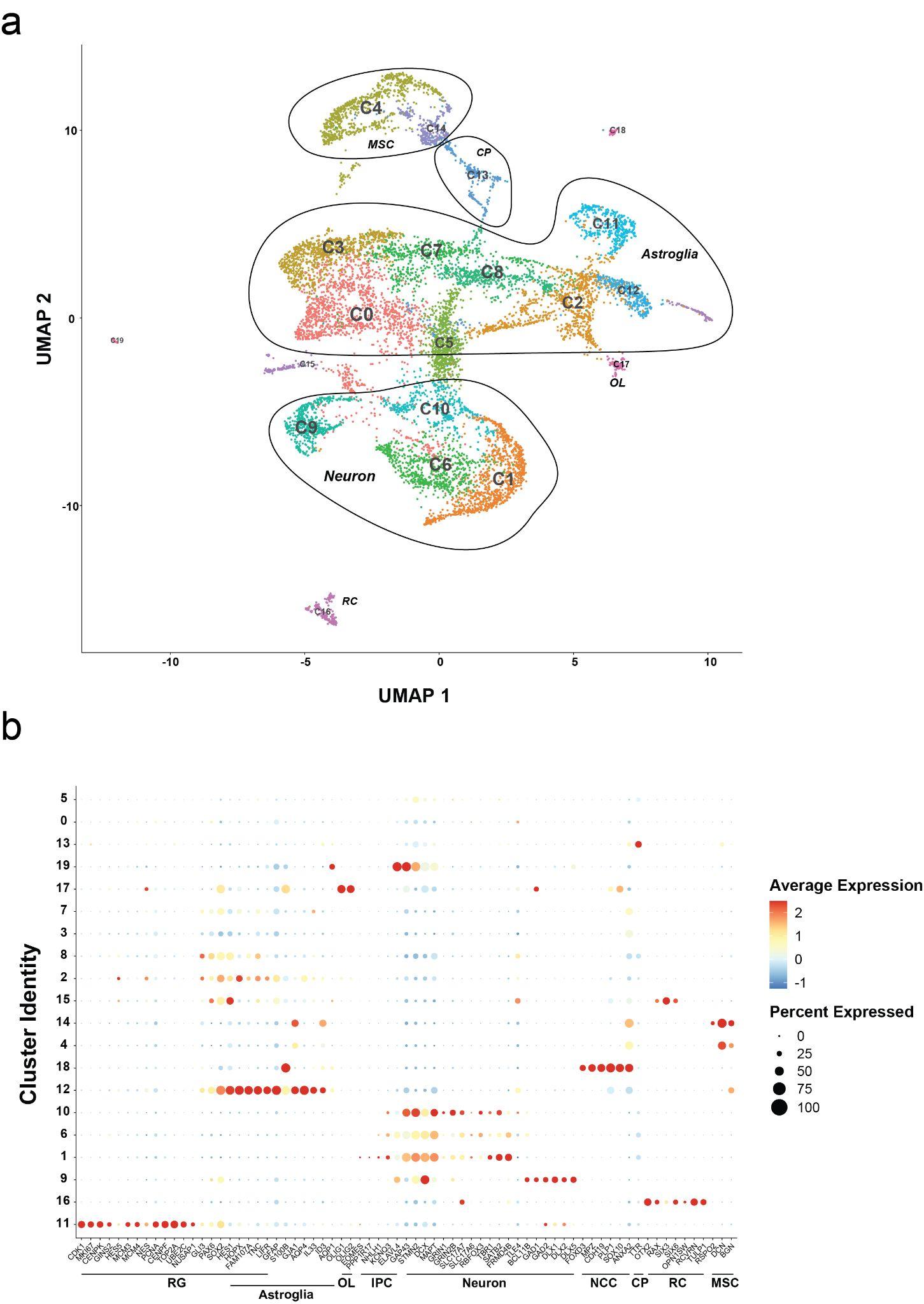
**

**Figure S2. Single-cell RNA sequencing (scRNA-Seq) reveals multiple cell types in control hCOs.** (a) UMAP plot of pooled iPSC and ESC hCO scRNA data revealed clusters linked to the expression of specific cellular markers. (b) DotPlot visualization of the expression of different cell type-specific marker genes for all clusters depicted in panel a. Abbreviations: RG, radial glia; OL, oligodendrocytes; IPC, intermediate progenitor cells; NCC, neural crest cells; CP, choroid plexus; RC, retinal cells; MSC, mesenchymal stem cells.

**
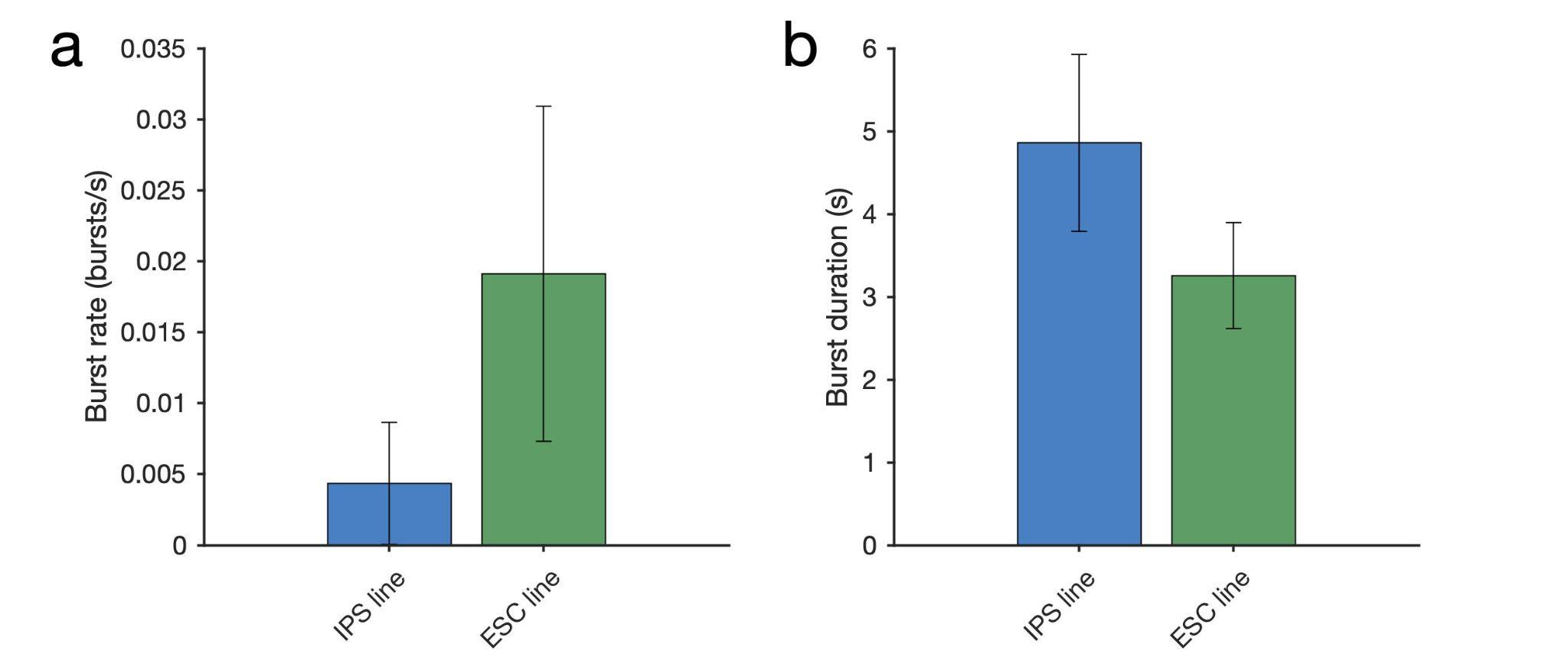
**

**Figure S3. Comparison of burst rate and burst duration across the IPSC and ESC lines.** (a) hCO burst rate, measured as the number of bursts per second, varied significantly between the iPSC and ESC lines. ESC-derived hCOs showed a higher burst rate compared to the iPSC line. (b) The burst duration also differed between the iPSC and ESC line. hCOs of the ESC line showed shorter bursts, compared to the iPSC line.

**
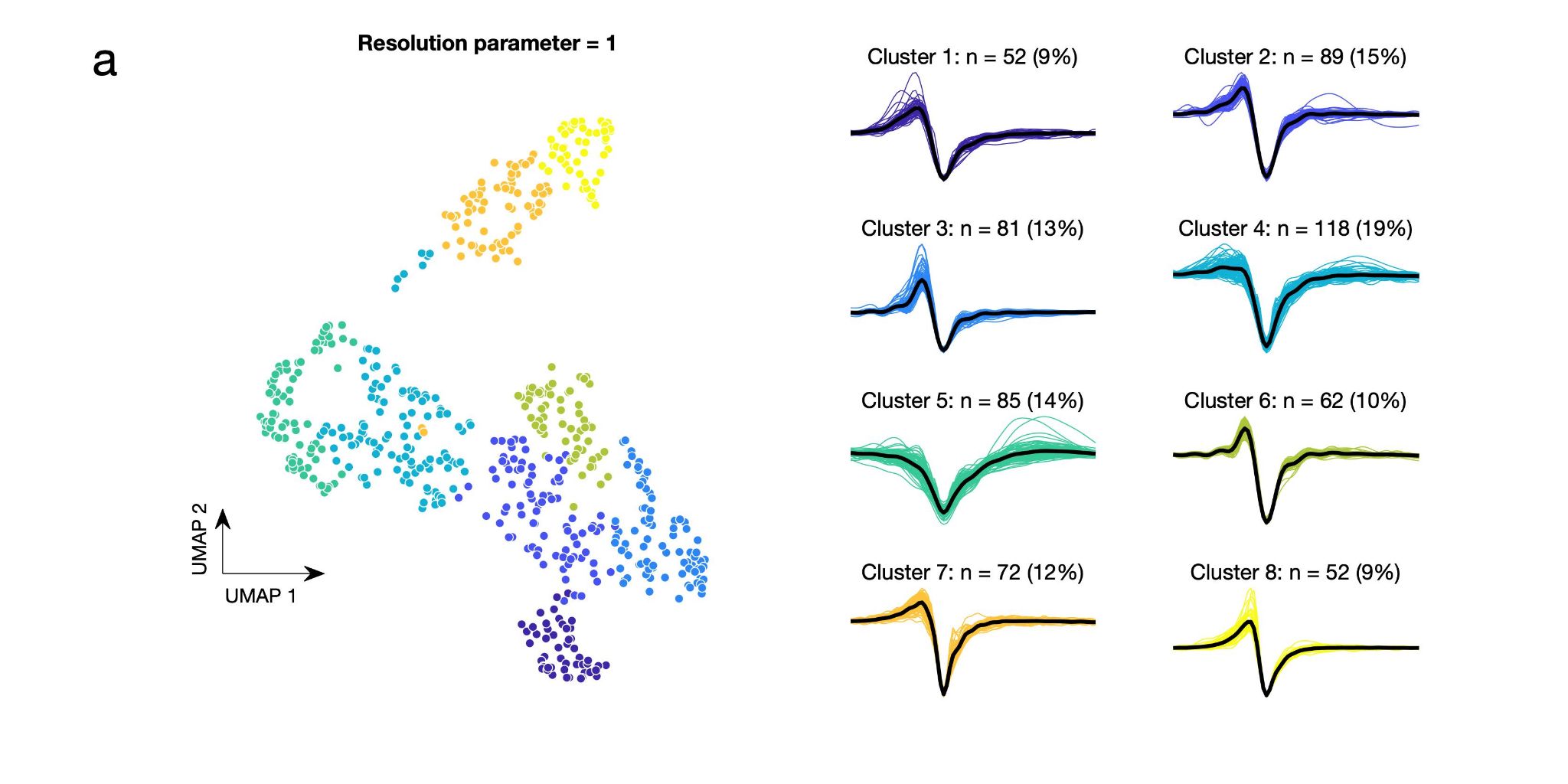

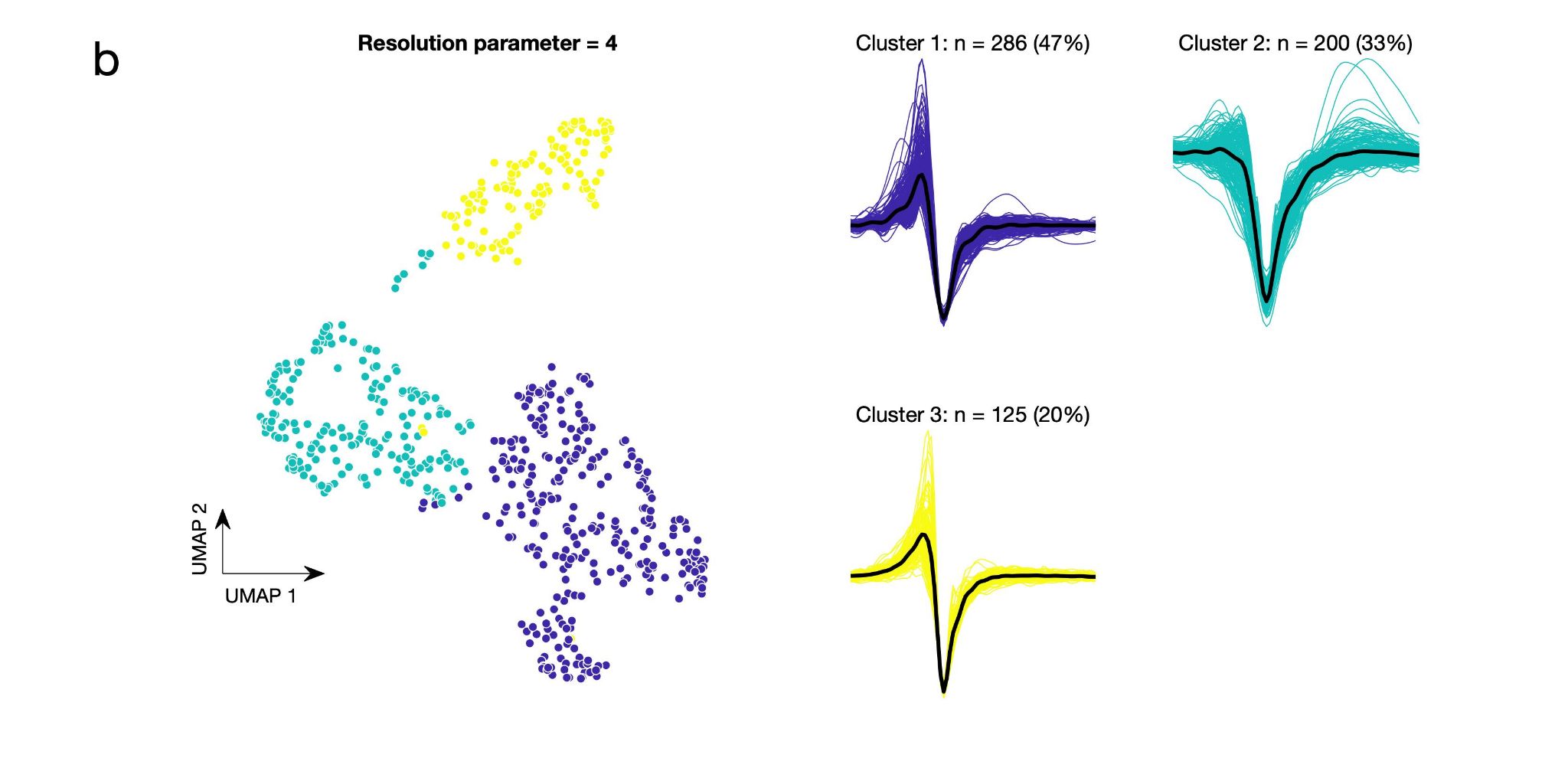
**

**Figure S4. Comparison of different resolution parameters for the WaveMap clustering analysis.** a) UMAP projection plot resulting from a WaveMap clustering analysis with 611 units. Each dot is colored according to the Louvain community detection, here for a resolution parameter of 1. On the right, the normalized waveform signals for each cluster are plotted. Panel b) shows the same plot as in a), however, for a resolution parameter of 4. For a resolution parameter of 4, the Louvain community detection resulted in three clusters.

**
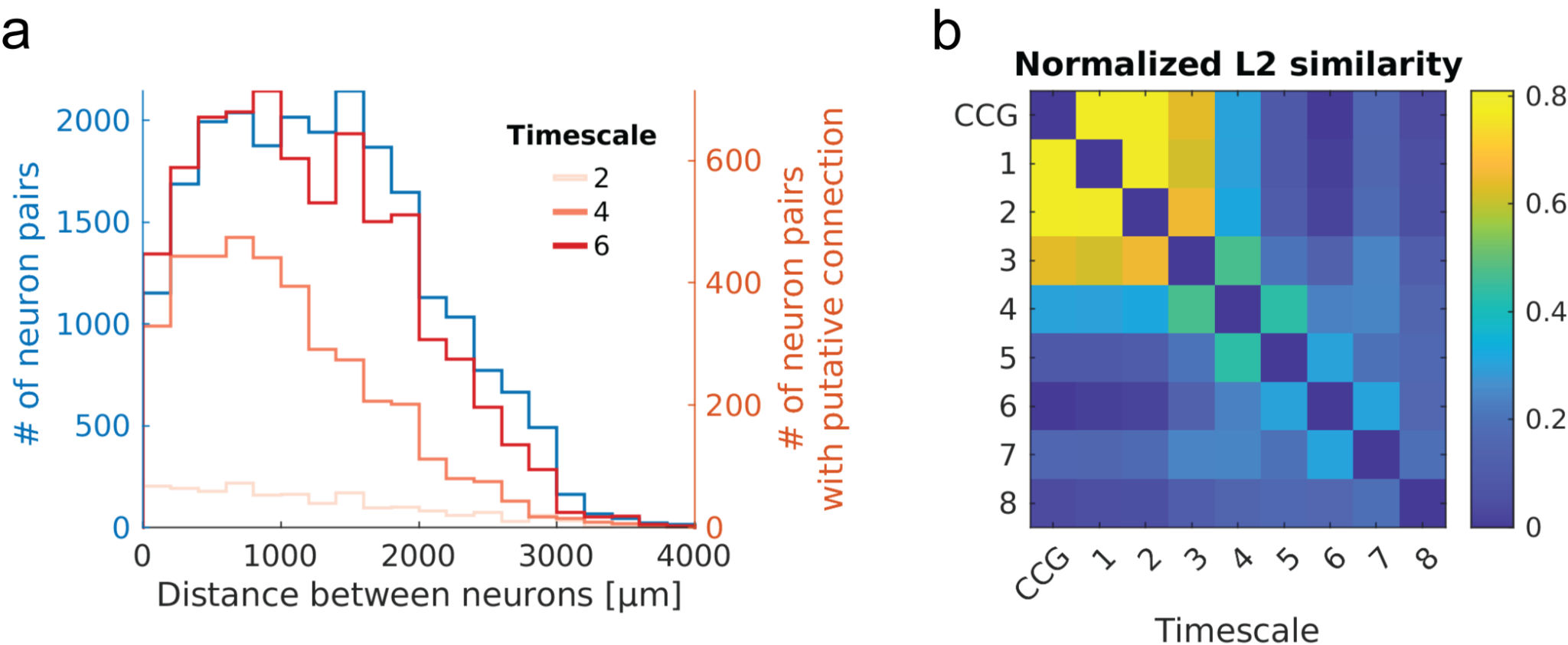
**

**Figure S5. Comparison of transfer entropy-based functional connectivity inference across different time scales.** a) Inter-neuronal distances as a function of the number of significant connections, inferred with the transfer entropy (TE) method at different time scales. Lines colored in different shades of red indicate TE connectivity inference results at time scales 2, 4 and 6 (see Supplemental Table 1 for details on each time scale). The number of significant connections increased with the time scale length, while the distance dependency of connections became less apparent. b) The similarity between TE connectivity matrices inferred at different time scales was quantified by the normalized Euclidean distance. The displayed similarity matrix was calculated by subtracting the normalized Euclidean distance from 1, resulting in values between 0 and 1. The diagonal was set to 0 for visual clarity. The connectivity, inferred with the cross-correlogram (CCG)-based method, was more similar to TE connectivity at shorter time scales; the similarity between CCG and TE connectivity decreased strongly for longer time scales.

| **Time Scale** | **Bin Size [ms]** | **Source Delay** | **Source Window [bins]** | **Source Window [ms]** | **Jitter Window** |
| --- | --- | --- | --- | --- | --- |
| 1 | 1 | 0 | 0-3 | 0.05-3 | 7 |
| 2 | 1.6 | 1 | 1-4 | 1.6-6.4 | 11.2 |
| 3 | 3.5 | 1 | 1-4 | 3.5-14 | 24.5 |
| 4 | 7.5 | 1 | 1-4 | 7.5-30 | 52.5 |
| 5 | 16.15 | 1 | 1-4 | 16.15-64.6 | 113.05 |
| 6 | 34.8 | 1 | 1-4 | 34.8-139.2 | 243.6 |
| 7 | 75 | 1 | 1-4 | 75-300 | 525 |
| 8 | 161.6 | 1 | 1-4 | 161.6-646.4 | 1131.2 |

**Supplemental Table 1. Parameters describing the different time scales.** The bin size was logarithmically increased to cover a larger spectrum of different relevant time scales. The source delay was 0 at time scale 1 to capture extreme short-latency interactions, and left at 1 for time scales 2-10. The target history was kept constant at 1 for all time scales (not shown).
